# Neuromuscular components of Apgar score in predicting delivery room respiratory support

**DOI:** 10.1038/s41390-024-03281-6

**Published:** 2024-05-20

**Authors:** Manoj Biniwale

**Affiliations:** Department of Pediatrics, Keck Medical School of USC, 1200 N State Street, Los Angeles, CA 90033 USA

## Commentary

Newborn wellbeing is documented by use of Apgar scores after every delivery of the infant all over the world. Scores developed by Dr Virginia Apgar in 1952 are still considered as the gold standard for the assessment of newborns at 1 and 5 min of age by both the American College of Obstetricians and Gynecologists, and the American Academy of Pediatrics.^1^ Five elements of Apgar scores namely heart rate, respiration, color, activity (muscle tone) and grimace (reflexes) constitute equal points ranging from 0 to 2 leading to maximum score of 10 points. Neonatal resuscitation program typically focuses on heart rate, breathing and color (oxygen saturations) for steps in performing resuscitation. There is less emphasis given for grimace and activity during decision making at the time of resuscitation. Even though activity and grimace essentially reflect neuromuscular response of the infant with tone and reflexes respectively its utility has been limited especially in preterm infants. While assessment of heart rate, breathing and color are easy to interpret with placement of cardiac monitor and pulse oximeter in preterm infants there is significant variability between delivery room personnel to interpret scoring of activity and grimace as compared to term infants.^2^

Majority of very preterm infants require some form of resuscitation ranging from oxygen through mask or nasal cannula, continuous positive airway pressure (CPAP), positive pressure ventilation (PPV) to intubation with advanced cardiopulmonary resuscitation. It is of utmost importance to provide the optimum respiratory support in these preterm infants to avoid further morbidities which may lead to long term consequences. Current delivery room decisions while caring for preterm infants are based on the response to initial steps of resuscitation as well as individual experience of personnel leading resuscitation and local practice standards. Predicting stability in these infants is the highest priority in order to avoid unnecessary escalation of support. Even if the decisions to provide or escalate respiratory support are made based on heart rate and oxygen saturations in reality getting accurate heart rate and oxygen saturations in the first minutes of life in very preterm infants may not be always feasible with pulse oximetry.^3^ The study by Tuttle et al in this edition of Pediatric Research have addressed the concerns in predicting interpersonal variability and assessing need for respiratory support while focusing on two of the least relied upon components of Apgar score namely grimace and activity.^4^

The authors hypothesize that initial grimace and activity reported in Apgar scores can predict reliably what respiratory support is required for these very preterm infants to successfully achieve stabilization. The study uses videos of prerecorded resuscitation conducted from previous study and quality improvement measures to calculate grimace and activity scores at early stages of resuscitation in very preterm infants. It is interesting to note that seven neonatal consultants were blinded to report the grimace and activity scores on the video clips prior to receiving the respiratory support. The videos were condensed to get immediate reflection of scores from the blinded personnel. Additionally, the authors deleted audios from all clips to avoid any bias. Assessors scored the video clips only once and median scores were considered for analyses. Further, the scores for grimace and activity were combined, and infants were separated in two groups. The infants were labeled non-vigorous if combined score was <2 and vigorous if score was ≥2. Outcome of respiratory support was divided into three categories of no respiratory support/CPAP, PPV for longer than 15 seconds and attempts for intubations. Additionally, the team assessed interobserver variability for scoring these two components of Apgar score.

Authors did not find any significant difference in the requirement of respiratory support when they analyzed activity and grimace as individual factors. On the other hand, when the scores were combined together, the infants labeled as non-vigorous were at significantly higher risk for requiring greater level of respiratory support. Further, these infants were less likely to be stabilized by CPAP alone. Another interesting finding was that initially there was some correlation between lower heart rate in non-vigorous infants. Over time authors noted a poor correlation between non-vigorous infants when compared to heart rate and oxygen saturations. Decision making for the respiratory support in the delivery room is more complex than simply following the algorithm especially in extremely preterm infants. One must weigh in the facts that even if heart rate and oxygen saturations are relatively within the range whether it is sustainable for the non-vigorous infants to achieve a longer term stabilization without needing additional respiratory support. Additionally, immaturity of lungs plays a significant role due to variability in surfactant production.

Heart rate, respiration and color typically assess cardiorespiratory wellbeing of the newborn infants whereas grimace and activity of Apgar scores typically assess neuromuscular response. Thus combining these two neuromuscular components may give better perception of overall infant wellbeing. It makes sense that the neuromuscular factors would predict the need for initial respiratory support independent of heart rate and oxygen saturations as shown in the present study. There are several causes of having abnormal tone and reflexes at birth. Predominant prenatal causes include compromised blood flow through placenta due to maternal hypertension or abruption. Maternal medications such as magnesium sulfate or administration of general anesthesia can compromise the tone in preterm infants. Additionally, infant causes typically include sepsis, metabolic derangements and neurological insult among other.^1,5^ The present study only includes few of these factors to differentiate the etiology of having low grimace and activity scores, and their effects on the outcomes.

Video recordings of neonatal resuscitation is practiced at several institutions all over the world mainly to review local practices and improve the performance of resuscitation.^6^ The video recordings have been also used in clinical research studies to get accurate time points for the resuscitation and used as a tool in the training to assess adherence with algorithms and guidelines as well as technical, cognitive and behavioral skills.^7^ The present study used small clips of videos recorded during the resuscitation of preterm infants. The clips shown to the blinded consultants were only 5–20 s in length. It is interesting to note that even the brief clips were assessed accurately by these blinded consultants to predict the decision making for the need of respiratory support during the resuscitation. Further, it is impressive to see a very little variability among the consultants with most of them showing agreement to similar scores of grimace and activity. One may argue that the scoring consultants were from only two large institutions compared to previous studies showing more variability among neonatologists from several centers with different backgrounds.^2,8^

Overall this study brings very unique aspects of Apgar scoring components. Activity and grimace are typically assigned in retrospect for the majority of very preterm resuscitations. These two components are generally not considered as priority and ignored for initialing resuscitation measures or assessing response. The present study may lead to more clinical research questions on how to interpret grimace and activity, and whether these two factors play more role than we think especially in very preterm infants. Previous studies have shown a low predictability of Apgar scores in assessing long term neurodevelopmental outcomes in preterm infants which was partly due to variability in scoring.^9^ Recently using machine learning algorithms researchers have assessed various factors including all components of Apgar scores that play roles in robust prediction of survival in extremely preterm infants.^10^ One may use the resuscitation videos along with pertinent clinical information into the artificial intelligence models which may improve accuracy and limit variability in predicting the need for respiratory support in preterm infants. Additionally, whether neuromuscular responses at birth have any impact of future general movement assessment is remained to be studied. Researchers may also assess correlation between the response to resuscitation using brain oxygen levels with near infrared spectroscopy and initial neurological responses recorded through grimace and activity. Further studies may focus on assessing long term consequences of having low scores in activity and grimace to independently predict neurodevelopmental outcomes.
